# Comparison of the Novel Oral Anticoagulants Apixaban, Dabigatran, Edoxaban, and Rivaroxaban in the Initial and Long-Term Treatment and Prevention of Venous Thromboembolism: Systematic Review and Network Meta-Analysis

**DOI:** 10.1371/journal.pone.0144856

**Published:** 2015-12-30

**Authors:** A. T. Cohen, M. Hamilton, S. A. Mitchell, H. Phatak, X. Liu, A. Bird, D. Tushabe, S. Batson

**Affiliations:** 1 Guy’s and St Thomas’ Hospitals, King’s College, London, United Kingdom; 2 BMS, Princeton, United States of America; 3 Abacus International, Bicester, United Kingdom; 4 Pfizer, New York, United States of America; 5 Pfizer, Walton Oaks, United Kingdom; 6 TUSH-D UK LTD, Birmingham, United Kingdom; Maastricht University Medical Center, NETHERLANDS

## Abstract

**Background:**

Anticoagulation with low molecular weight heparin and vitamin K antagonists is the current standard of care (SOC) for venous thromboembolism (VTE) treatment and prevention. Although novel oral anti-coagulants (NOACs) have been compared with SOC in this indication, no head-to-head randomised controlled trials (RCTs) have directly compared NOACs. A systematic review and network meta-analysis (NMA) were conducted to compare the efficacy and safety of NOACs for the initial and long-term treatment of VTE.

**Methods:**

Electronic databases (accessed July 2014) were systematically searched to identify RCTs evaluating apixaban, dabigatran, edoxaban, and rivaroxaban versus SOC. Eligible patients included adults with an objectively confirmed deep vein thrombosis (DVT), pulmonary embolism (PE) or both. A fixed-effect Bayesian NMA was conducted for outcomes of interest, and results were presented as relative risks (RR) and 95% credible intervals (Crl).

**Results:**

Six Phase III RCTs met criteria for inclusion: apixaban (one RCT; n = 5,395); rivaroxaban (two RCTs; n = 3,423/4,832); dabigatran (two RCTs; n = 2,539/2,568); edoxaban (one RCT; n = 8,240). There were no statistically significant differences between the NOACs with regard to the risk of ‘VTE and VTE-related death. Apixaban treatment was associated with the most favourable safety profile of the NOACs, showing a statistically significantly reduced risk of ‘major or clinically relevant non-major (CRNM) bleed’ compared with rivaroxaban (0.47 [0.36, 0.61]), dabigatran (0.69 [0.51, 0.94]), and edoxaban (0.54 [0.41, 0.69]). Dabigatran was also associated with a significantly lower risk of ‘major or CRNM bleed’ compared with rivaroxaban (0.68 [0.53, 0.87]) and edoxaban (0.77 [0.60, 0.99]).

**Conclusions:**

Indirect comparisons showed statistically similar reductions in the risk of ‘VTE or VTE-related death for all NOACs. In contrast, reductions in ‘major or CRNM bleed’ for initial/long-term treatment were significantly better with apixaban compared with all other NOACs, and with dabigatran compared with rivaroxaban and edoxaban. Results from the current analysis indicate that the NOACs offer clinical benefit over conventional therapy while highlighting relative differences in their bleeding profile.

## Introduction

Venous thromboembolism (VTE) comprises deep vein thrombosis (DVT) and pulmonary embolism (PE). VTE is associated with a high risk of recurrence after a first event. On the cessation of anticoagulation therapy, approximately 10% of patients with VTE experience a recurrence within a year after the first event [1, 2] and 30% have a recurrence within 10 years [2, 3] and the risk of recurrence is dependent on several factors [4]. Globally, VTE represents a substantial personal and economic burden [5, 6]; yet it is a preventable cause of long-term morbidity and mortality. VTE is associated with long-term, clinically significant complications, including post-thrombotic syndrome, reported in up to 50% of patients with VTE [7], and chronic thromboembolic pulmonary hypertension in up to 4% of patients with PE [8]. Lastly, VTE is associated with substantial mortality [9, 10]; the all-cause mortality rate is reported to be approximately 5% after 1 year in the VTE population [11].

Effective treatment of VTE relies on a balance between the prevention of recurrence and the incidence of bleeding complications [12]. In general, clinical guidelines for the treatment of VTE recommend subcutaneous low-molecular-weight-heparin (LMWH), as well as fondaparinux [13–15], followed by a vitamin K antagonist (VKA) [13]. Both LMWH and VKAs (such as warfarin, acenocumerol or phenprocoumon) are associated with a risk of (potentially fatal) bleeding [16, 17]. Furthermore, LMWHs may be inconvenient for patients as they can only be administered subcutaneously and VKAs require monitoring for optimal dosing [16] and carry the risk of drug interactions. Novel oral anticoagulants (NOACs) were developed to offer efficient anticoagulation while eliminating the need for monitoring. The four main NOACs currently being studied/approved for the treatment of VTE are rivaroxaban, edoxaban, and apixaban (all direct Factor Xa inhibitors), and dabigatran (a direct thrombin inhibitor). Of these, apixaban, dabigatran, and rivaroxaban are now approved for the treatment of VTE as well as for the prevention and treatment of DVT and PE in patients undergoing orthopaedic surgery, both in the EU and the USA. Edoxaban is currently approved in Japan for the prevention of VTE after major orthopaedic surgery and is approved in the USA (and has received a positive opinion from the European Committee for Medicinal Products) for the treatment and secondary prevention of VTE in a non-surgical population. Compared with VKAs, NOACs offer rapid onset of action, fixed dosing, no known food effects, fewer drug interactions, no requirement for routine monitoring of fixed doses, and a short offset period [18].

The current evidence base for the efficacy and safety of NOACs does not include any head-to-head trials directly comparing the different NOACs [19–24]. It is important to assess the relative clinical value of NOACs from health care providers’ and payers’ perspective. Therefore, our objective was to conduct a systematic review and network meta-analysis (NMA) comparing the efficacy and safety of NOACs for the initial and long-term treatment and secondary prevention of VTE.

## Methods

### Systematic Review

A systematic review protocol was written to define all aspects of the review prior to commencement. The pre-defined inclusion criteria are reported in Table 1.

**Table 1:** Inclusion criteria for the systematic review.

Population	Adult patients (≥18 years of age) with an objectively confirmed symptomatic VTE (DVT and/or PE), who were receiving initial/long-term treatment following an acute VTE event. Patients receiving extended treatment for secondary prevention of VTE were not eligible for inclusion†.
Interventions	Treatments of interest include the following NOACs:
	• Apixaban
	• Dabigatran
	• Rivaroxaban
	• Edoxaban
Comparator	Warfarin/VKA
Outcomes	Studies were included if they reported ≥ 1 of the following priority outcomes
	• Recurrent VTE and VTE-related death
	• Major bleeding
	• CRNM bleeding
	• Major or CRNM bleeding
	• All-cause mortality
Study design	Prospective, phase III RCTs, with no restriction on randomisation procedure: double-blind or open label
Date and language of publication	No date restriction; only publications in English language were included

Abbreviations: CRNM, clinically relevant non-major bleeding; DVT, deep vein thrombosis; PE, pulmonary embolism; RCT, randomised controlled trial; VKA, vitamin K antagonist; VTE, venous thromboembolism

†The most recent ACCP guidelines [25]define long-term therapy as either complete treatment of an acute VTE episode or prevention of additional VTE events unrelated to the primary event. Extended treatment refers to anticoagulation continued post 3 months without any scheduled completion date.

### Literature searches

The data sources to identify published studies and ongoing (unpublished) studies included: electronic databases searched on 14^th^ July 2014 (Embase 1980 onwards; MEDLINE® In-Process & Other Non-Indexed Citations; OVID MEDLINE 1946 onwards; Cochrane Library (NHS EED), 1968 onwards) and conference proceedings from 2011 to 2013 (American Society of Hematology, International Society on Thrombosis and Haemostasis, European Hematology Association). Two reviewers working independently screened the titles and abstracts in addition to the full publications against the pre-specified criteria. Any disagreements were resolved through discussion until a consensus was reached, or the involvement of a third reviewer. Double data extraction of eligible outcome data was conducted by two researchers with any disputes referred to a third party.

### Quality assessment

The quality of RCTs was assessed according to the methodology checklist detailed in Appendix D of the NICE Guidelines Manual 2009 [26]. The likelihood of selection, attrition, and detection and performance bias was assessed by two reviewers working independently. As above, disagreements were resolved by discussion and/or involvement of a third reviewer.

### Network meta-analysis

NMA was used to obtain comparisons between all treatments. WinBUGS software (MRC Biostatistics Unit, Cambridge, UK) was used to conduct a Bayesian NMA. Models were run for 50,000 iterations to calculate the point estimate of comparisons between treatments. The treatment effect was evaluated in terms of relative risk. The point estimate represented the median of the posterior distribution with an associated 95% credible interval (Crl) taken from between the 2.5^th^ and 97.5^th^ percentiles of the distribution of the calculated data. The Crl is the Bayesian equivalent of a confidence interval (CI).

Both fixed- and random-effect models were employed. However, data are presented from the fixed-effect model only as this model gave the lowest deviance information criterion (DIC) compared with the random-effects model. In addition the evidence networks contained too few studies to provide a feasible and precise estimate of the between study variance in the random-effects model [27]. Information on treatment ranking can give misleading results when the evidence network is sparse and therefore these data are not presented and emphasis is placed on the relative treatment effects and their uncertainty.

### Data sources

Analyses were performed on a dichotomous dataset comprising the number at risk and the number of events of an outcome of interest. The outcomes of interest were: (a) VTE and VTE-related death. This was a composite efficacy endpoint, consisting of reported events of DVT, fatal or non-fatal PE and VTE-related death; (b) major bleeding; (c) clinically relevant non-major (CRNM) bleeding; (d) major or CRNM bleeding; (e) all-cause mortality. Analyses of efficacy outcomes considered the number of events in an intention-to-treat (ITT) study population as defined by each study, whereas analyses of safety outcomes were based on the reported safety population.

Additional analyses were conducted for non-fatal PE, DVT, VTE-related death (i.e. death related to any VTE event, or where VTE could not be ruled out as a cause of death), intracranial haemorrhage, other major bleeding, other deaths, and overall treatment discontinuation.

### Data assumptions for NMA

There were no data reported for CRNM bleeding in the RE-COVER [24] and RE-COVER II [22] publications. To obtain event data for this outcome from the trials, the reported ‘major bleeding’ outcome data were subtracted from the reported ‘major or CRNM bleeding’ data. Additional data assumptions were required for the other secondary outcomes, and these are reported in the supporting information.

Sensitivity analyses were conducted for each outcome, substituting data from the individual RE-COVER [24] and RE-COVER II [22] trials with data from the pooled analysis of the RE-COVER and RE-COVER II trials [22]. The pooled analysis was conducted after results from both trials were available and was based on further adjudication of events reported after publication of the RE-COVER trial [24]. A full explanation of the differences between the datasets is provided by the authors [22].

## Results

**Fig 1:**
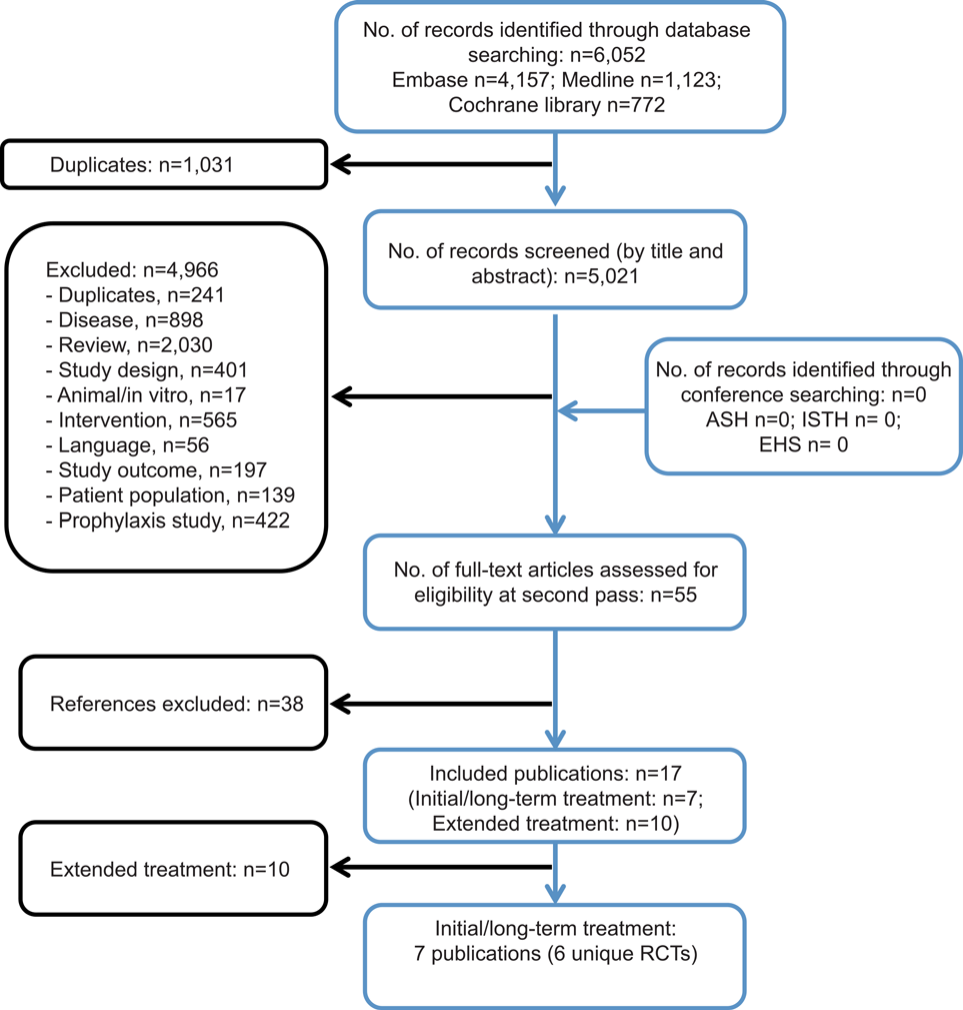
Systematic review flow diagram. The flow diagram indicates inclusion and exclusion of publications at each stage of the systematic review process.

The initial electronic database search (accessed July 14th 2014) identified 6,052 articles, of which 5,021 were screened (after removal of duplicates). In total, 4,966 publications were excluded on the basis of title and abstract. On application of the review inclusion criteria to the 55 full-text papers, a further 38 were excluded. Therefore 17 publications met the inclusion criteria and were included in the systematic review. Ten publications reported on the extended treatment of VTE in patients who had received prior initial treatment and were excluded from the current meta-analysis. Therefore seven publications detailing six unique RCTs reported relevant outcome data for the initial and long-term treatment of VTE (Fig 1) and were included in the meta-analysis: AMPLIFY [21], RE-COVER [24], RE-COVER II [22], Hokusai-VTE [23], and pooled data [28] from the EINSTEIN DVT [19] and the EINSTEIN PE [20] trials. The EINSTEIN pooled data [28] were used in the NMA to ensure that the source data covered a general VTE population, rather than the respective DVT and PE populations used in the individual EINSTEIN trials. This approach was taken to ensure comparability of the data used and to maximize the statistical power of our analysis.

**Fig 2:**
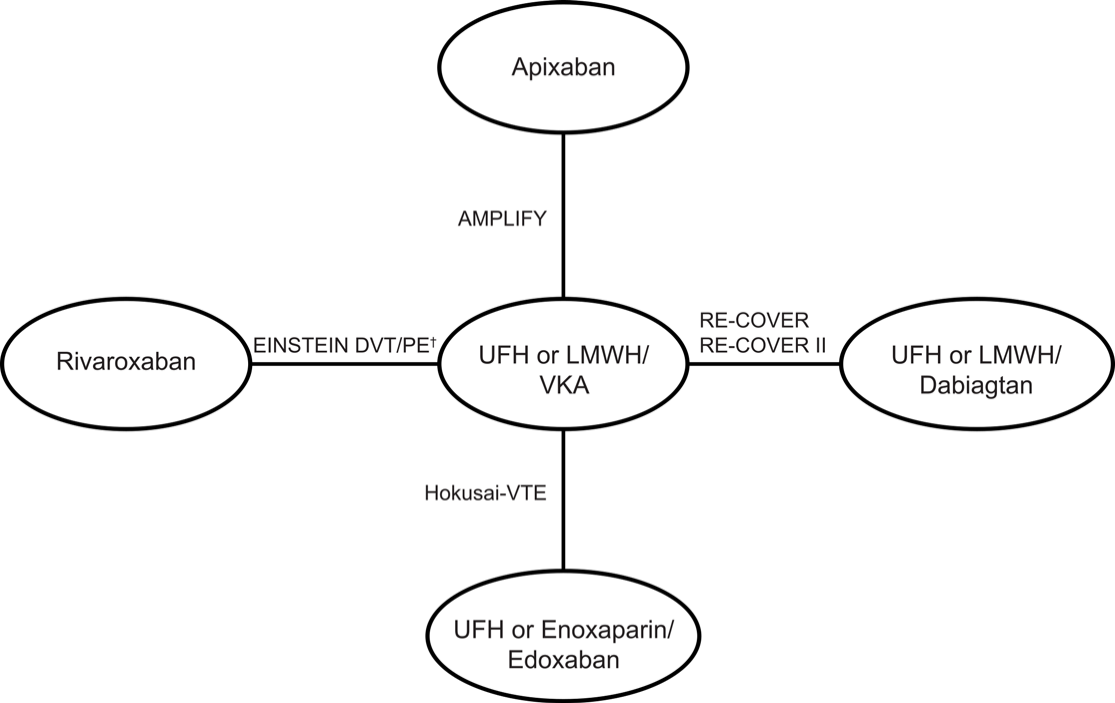
Network of evidence for the meta-analysis. †Primary and sensitivity analyses used pooled data from the EINSTEIN DVT and EINSTEIN PE trials [28].

Table 2 summarises the characteristics of each study. The reported mean age of patients and the percentage of female patients were similar across trials. All studies were judged to be of good quality (S1 Table) although there were some differences between the trials in terms of study design and patient characteristics: AMPLIFY [21], Hokusai-VTE [23], RECOVER [24] and RECOVER II [22] were double-blind, whereas the EINSTEIN DVT [19] and EINSTEIN PE [20] trials were open-label. AMPLIFY [21] reported 89.8% of patients as having an unprovoked VTE, whereas the pooled analysis of the EINSTEIN DVT/PE studies [28] and Hokusai-VTE [23] reported 63.5% and 65.7%, respectively. This suggests some variation in the baseline risk of VTE between trials, as patients with an unprovoked VTE may have a higher risk of recurrence [29]. Finally, the EINSTEIN DVT [19] and EINSTEIN PE [20] trials had treatment periods of 3, 6 or 12 months (at discretion of clinician), Hokusai-VTE [23] had a maximum treatment duration of 12 months compared with a treatment duration of 6 months in the remaining trials [21, 22, 24]. Initial treatment (≥5 days) with an approved parenteral anticoagulant was required in the RE-COVER [24], RE-COVER II [22] (LMWH in the majority of patients [~90%]) and Hokusai-VTE studies [23] (enoxaparin or unfractionated heparin [UFH]) prior to receiving a NOAC or warfarin, in contrast to AMPLIFY [21] and the EINSTEIN studies [19] where treatments were administered as single regimens. There was variation in the definition of the primary efficacy outcome across the studies. The ‘VTE and VTE-related death’ endpoint comprised reported events of DVT, fatal or non-fatal PE, and VTE-related death. This was reported in the trial publications as follows: AMPLIFY [21]–adjudicated composite of recurrent VTE or death related to VTE. Recurrent VTE included fatal or non-fatal PE and DVT; RE-COVER [24]–composite of VTE or death associated with VTE; RE-COVER II [22]–VTE or VTE-related death; EINSTEIN DVT [19], EINSTEIN PE [20] and Hokusai-VTE [23]–composite of DVT and non-fatal or fatal PE. All studies reported a consistent definition of bleeding outcomes as defined by the International Society on Thrombosis and Haemostasis [30]. The network of trials used is shown in Fig 2. The raw data used in the NMA are presented in S2 Table.

**Table 2:** Summary of study characteristics.

Trial	Treatment, number randomised	Mean age (SD)	Female, %	Unprovoked VTE, %	Unprovoked VTE (trial level), %	Patients with cancer, %	Index DVT, %	Index PE, %	Index DVT/PE, %	Time on treatment
AMPLIFY [21] Double-blind RCT	Apixaban 10 mg BD/5 mg BD^†^ (n = 2,691)	57.2 (16.0)	41.7	89.8	89.8	2.5	65.0	25.2	9.4	6 months
	Enoxaparin/warfarin INR 2.0–3.0^‡^ (n = 2,704)	56.7 (16.0)	40.9	89.8	89.8	2.8	65.9	25.2	8.3	6 months
RE-COVER [24] Double-blind RCT	UFH or LMWH/dabigatran 150 mg BD^§^ (n = 1,274)	55.0 (15.8)	42.0	NR	NR	5.0	69.1	21.2	9.5	6 months
	UFH or LMWH/warfarin INR 2.0–3.0^¶^ (n = 1,265)	54.4 (16.2)	41.1	NR	NR	4.5	68.6	21.4	9.8	6 months
RE-COVER II [22] Double-blind RCT	UFH or LMWH/dabigatran 150 mg BD^§§^ (n = 1,279)	54.7 (16.2)	39.0	NR	NR	3.9	68.5	23.3	8.1	6 months
	UFH or LMWH/warfarin INR 2.0–3.0^¶¶^ (n = 1,289)	55.1 (16.3)	39.8	NR	NR	3.9	67.8	23.1	9.1	6 months
EINSTEIN DVT/ EINSTEIN PE pooled analysis [28] Open-label RCTs	Rivaroxaban 15 mg BD/20 mg OD^††^ (n = 4,151)	57.0 (17.0)	44.5	63.1	63.5	5.6	NR (41.7^¶¶¶^)	NR (58.3^¶¶¶^)	NR	3, 6 or 12 months^†††^
	Enoxaparin/VKA INR 2.0–3.0^‡‡^ (n = 4,131)	57.0 (16.8)	46.4	63.8	63.5	4.8	NR (41.6^¶¶¶^)	NR (58.4^¶¶¶^)	NR	3, 6 or 12 months^†††^
Hokusai-VTE [23] Double-blind RCT	Enoxaparin or UFH/Edoxaban 60 mg OD^§§§^ (n = 4,143)	55.7 (16.3)	42.7	65.9	65.7	9.2	59.9	30.1	10.0^‡‡‡^	Maximum of 12 months
	Enoxaparin or UFH/warfarin INR 2.0–3.0^§§§^ (n = 4,149)	55.9 (16.2)	42.8	65.4	65.7	9.5	59.5	30.7	9.8^‡‡‡^	Maximum of 12 months

Abbreviations: BD, twice daily; DVT, deep vein thrombosis; LMWH, low molecular weight heparin; NR, not reported; OD, once daily; PE, pulmonary embolism; RCT, randomised controlled trial; SD, standard deviation; VKA, vitamin K antagonist; VTE, venous thromboembolism

^†^Patients received 10 mg apixaban twice daily for the first 7 days, followed by 5 mg twice daily for 6 months.

^‡^Patients received enoxaparin 1mg/kg every 12 hours for ≥5 days and warfarin (adjusted to INR 2.0–3.0) begun concomitantly and continued for 6 months. Enoxaparin was discontinued when the INR was 2.0–3.0.

^§^Patients received initial treatment with an approved parenteral anticoagulant (UFH, 11.3%; LMWH, 89.4%; fondaparinux, 3.9%) for a median of 6 days (IQR 5–8) [continued for ≥5 days and until INR was 2.0–3.0] (single dummy phase). Patients then received dabigatran 150 mg twice daily for 6 months (‘double dummy phase).

^¶^Patients received initial treatment with an approved parenteral anticoagulant (UFH, 13.0%; LMWH, 90.7%; fondaparinux, 2.8%) for a median of 6 days (IQR 5–8) [continued for ≥5 days and until INR was 2.0–3.0] (single dummy phase). Patients also received warfarin on the day of randomisation (adjusted to INR 2.0–3.0) for 6 months (double dummy phase).

^††^Patients received rivaroxaban 15 mg twice daily for 3 weeks, followed by 20 mg once daily.

^‡‡^Patients received enoxaparin 1mg/kg twice daily for ≥5 days and either warfarin or acenocoumarol (adjusted to INR 2.0–3.0), started ≤48 hours post randomisation. Enoxaparin was discontinued when the INR was ≥2.0 for ≥2 consecutive days.

^§§^Patients received initial treatment with an approved parenteral anticoagulant (UFH, 15.5%; LMWH, 88.5%; fondaparinux, 2.5%) for a mean of 6.8 days (SD 3.4) [continued for ≥5 days and until INR was 2.0–3.0] (single dummy phase). Patients then received dabigatran 150 mg twice daily for 6 months (‘double dummy phase).

^¶¶^Patients received initial treatment with an approved parenteral anticoagulant (UFH, 16.1%; LMWH, 89.1%; fondaparinux, 1.6%) for a median of 7.1 days (SD 3.7) [continued for ≥5 days and until INR was 2.0–3.0] (single dummy phase). Patients also received warfarin on the day of randomisation (adjusted to INR 2.0–3.0) for 6 months (double dummy phase).

^†††^The intended treatment duration (3, 6, or 12 months) was determined by the treating physician before randomisation.

^‡‡‡^Patients with PE and concomitant DVT: edoxaban-treated patients (n = 410/1,650); warfarin-treated patients (n = 404/1669)

^§§§^All patients received initial therapy with open-label enoxaparin or UFH for ≥5 days. Edoxaban was started after discontinuation of initial heparin. Warfarin was started concurrently with the study regimen of heparin.

^¶¶¶^Within the pooled analysis, 1,731/4,151 (41.7%) patients were from the EINSTEIN-DVT study and received rivaroxaban and 1,718/4,131 (41.6%) patients received enoxaparin/VKA.

### NMA results

Due to the small number of studies in the network, the NMA was restricted to a fixed-effect model only. A random-effects model does not provide reliable estimates of the variation in between-study treatment effects when there are few studies in the network of evidence. Indeed, medical research guidelines recommend that calculations investigating heterogeneity should be based on a network of at least ten studies [31]. (i.e., only linear connections see Fig 2).

The NMA results for the primary outcomes of interest are reported in Table 3. The results for the comparison of the individual NOACs with LMWH/warfarin confirm the findings from the individual RCTs: there was no statistically significant difference in efficacy between the NOACs for ‘VTE or VTE-related death’. This was to be expected, due to the simple, not interconnected star shape of the evidence network (Fig 2), which results in the effect size for any treatment versus LMWH/VKA being driven by direct trial based results for the outcome in question. Our analysis shows a similar efficacy on mortality for the NOACs compared with conventional therapy, with apixaban being the only NOAC to show a significantly improved bleeding profile for all reported measures.

**Table 3:** Fixed-effect NMA results for primary outcomes of interest (significant results in bold).

Treatment comparison	RR (95% Crl)				
	VTE and VTE-related-death	Major or CRNM bleeding	Major bleeding	CRNM bleeding	All-cause mortality
Apixaban vs. VKA	0.83	**0.44**	**0.30**	**0.48**	0.79
	(0.59, 1.18)	**(0.35, 0.55)**	**(0.16, 0.53)**	**(0.38, 0.60)**	(0.52, 1.19)
Apixaban vs. rivaroxaban	0.93	**0.47**	0.55	**0.47**	0.82
	(0.59, 1.46)	**(0.36, 0.61)**	(0.27, 1.09)	**(0.36, 0.62)**	(0.50, 1.34)
Apixaban vs. dabigatran	0.76	**0.69**	**0.40**	0.80	0.79
	(0.47, 1.27)	**(0.51, 0.94)**	**(0.19, 0.81)**	(0.57, 1.12)	(0.44, 1.41)
Apixaban vs. edoxaban	1.01	**0.54**	**0.36**	**0.59**	0.75
	(0.63, 1.63)	**(0.41, 0.69)**	**(0.18, 0.69)**	**(0.45, 0.78)**	(0.47, 1.21)
Rivaroxaban vs. VKA	0.90	0.94	**0.55**	1.02	0.96
	(0.67, 1.20)	(0.82, 1.07)	**(0.37, 0.81)**	(0.88, 1.18)	(0.73, 1.27)
Rivaroxaban vs. dabigatran	0.82	**1.48**	0.73	**1.70**	0.96
	(0.52, 1.31)	**(1.15, 1.89)**	(0.40, 1.31)	**(1.28, 2.25)**	(0.59, 1.58)
Rivaroxaban vs. edoxaban	1.09	1.14	0.65	**1.26**	0.92
	(0.71, 1.69)	(0.94, 1.38)	(0.38, 1.09)	**(1.03, 1.55)**	(0.64, 1.33)
Dabigatran vs. VKA	1.09	**0.64**	0.76	**0.60**	1.00
	(0.76, 1.57)	**(0.51, 0.78)**	(0.48, 1.17)	**(0.47, 0.76)**	(0.66, 1.50)
Dabigatran vs. edoxaban	1.32	**0.77**	0.89	**0.74**	0.95
	(0.81, 2.16)	**(0.60, 0.99)**	(0.51, 1.57)	**(0.56, 0.98)**	(0.59, 1.52)
Edoxaban vs. VKA	0.83	**0.82**	0.85	**0.81**	1.05
	(0.60, 1.14)	**(0.72, 0.95)**	(0.59, 1.21)	**(0.70, 0.94)**	(0.82, 1.34)

Abbreviations: CrI, credible interval; CRNM, clinically relevant non-major; VKA, vitamin K antagonist; VTE, venous thromboembolism.

With regard to a head-to-head comparison of the NOACs, apixaban was associated with a significantly lower risk of ‘major or CRNM bleeding’ compared with dabigatran, edoxaban, or rivaroxaban. Apixaban also had a significantly lower risk of major bleeding and CRNM bleeding compared with dabigatran and rivaroxaban, respectively, and of both outcomes compared with edoxaban. Rivaroxaban was associated with a significantly higher risk of CRNM bleeding compared with dabigatran and edoxaban, and ‘major or CRNM bleeding’ compared with dabigatran. Finally, dabigatran treatment was associated with a significantly lower risk of ‘major or CRNM bleeding’ or CRNM bleeding compared with edoxaban. There were no other statistically significant differences between the NOACs for the outcomes reported in Table 3 (data for the inverse treatment comparisons are presented in S4 Table).

Results for other outcomes of interest (non-fatal PE, DVT, VTE-related death, intracranial bleeding, other major bleeding, other death, and overall treatment discontinuation) are presented in S3 Table. When comparing the NOACs, these results show a significantly decreased risk of ‘other major bleeding’ for apixaban vs all three comparators and for rivaroxaban vs. edoxaban. There was also a significantly reduced risk of treatment discontinuation for rivaroxaban vs edoxaban and dabigatran but not vs apixaban.

The results of the sensitivity analysis (S5 Table and S6 Table) using data from the pooled RE-COVER trials [22] support the findings observed in the primary analysis.

## Discussion

The clinical improvement observed with the use of NOACs may reduce the considerable burden associated with VTE. This burden comprises recurrent VTE events [32], high mortality rates [33], long-term complications [7], management of bleeding events, and issues regarding treatment with VKAs (the current standard of care [SOC]). NOACs could potentially mitigate this burden in terms of reduced bleeding risk, no requirement for monitoring, and a reduced potential for food/drug interactions, compared with VKAs. However, although clinical guidelines have been slow to recommend the use of NOACs, due to the relative paucity of clinical data [13, 15], this is changing [25, 34]. Only a small number of large-scale, high quality Phase III trials comparing NOACs with VKAs or LMWH have been published to date and there are no direct comparisons to allow an assessment of the relative efficacy and safety of the NOACs.

The current systematic review identified seven publications (six unique clinical trials and a pooled analysis of two trials) comparing four different NOACs (apixaban, dabigatran, edoxaban, and rivaroxaban) with warfarin and/or LMWH [19–24, 28]. Overall the quality of the trials was considered to be good (based on study size and design), although the RE-COVER II publication did not provide sufficient information for assessment (S1 Table). The two EINSTEIN studies [19] were open-label, whereas the remaining four studies were double-blind. Although in general double-blind RCTs are considered to be less subject to potential bias compared with open-label studies, issues such as consistency on endpoint assessment and reporting may have a larger impact on the internal/external validity of trial results for studies assessing oral anticoagulation therapies [35]. There were a number of limitations to our analysis, primarily the fact that although the methodology employed for the NMA is robust and validated [27], any indirect comparison is subject to potential bias not present in a direct head-to-head comparison. In addition, both the systematic review and NMA are limited by the small number of available studies. Furthermore, there were some differences in the patient baseline characteristics (percentage of patients with unprovoked VTE, percentage of patients with index DVT/PE event) and the trial methods (treatment duration, definition of outcomes, blinding status), which may impact the comparability of the reported data. However, while these differences might present a challenge to the similarity assumption between studies, it may reflect the external validity of these results as this variation in patient populations is more likely to reflect real-world practice.

Several previous meta-analyses reporting on the relative efficacy of the NOACs in this indication have been published [36–38]. The current analysis reports results for additional outcomes of interest and employs a Bayesian methodology. Data from the AMPLIFY study [21], the pooled analysis of the EINSTEIN trials [28], Hokusai-VTE [23], and full publication of the RE-COVER II RCT [22] were not available at the time of the analysis reported by Fox et al [36]. Data for apixaban was restricted to the phase II dose-ranging Botticelli study [39] which randomised 520 patients compared with AMPLIFY [21], which enrolled 5,395 patients. The reported indirect comparison was restricted to an assessment of the relative safety and efficacy of dabigatran and rivaroxaban only (recurrent VTE, major bleeding, and all-cause mortality). No statistically significant differences were reported between the two treatments which is consistent with the results from the current study (Table 3).

Four further recently published meta-analyses [37, 38, 40, 41] have included data from the Hokusai-VTE RCT [23] as did the current analysis. The primary focus of the van der Hulle meta-analysis [38] was a comparison of the class effect of NOACs compared with VKAs (again data from the RE-COVER II study and the associated pooled analysis [22] were excluded). The Mantha and Ansell [41] and Kang and Sobieraj [37] publications reported results for four and five efficacy/safety outcomes respectively and both analyses employed Bucher indirect comparison methodology. The reported results confirm the significantly reduced risk of major bleeding for apixaban compared with dabigatran and edoxaban as seen in the current analysis. Finally the meta-analysis by Castellucci [40] included a comparison of individual NOACs with UFH, LMWH, and fondaparinux (all in combination with VKA) or LMWH alone. Both apixaban and rivaroxaban were associated with a significantly lower risk of major bleeding compared with LMWH/VKA, the current SOC [40]. This reduction in the risk of major bleeding may be of particular clinical importance. There is increasing evidence to suggest that major bleeding in patients with VTE is not simply a benign event with no clinical consequences, but is associated with both increased morbidity and mortality [42–44].

With regard to the relative efficacy and safety of the NOACs, the current NMA indicates that although the NOACs report a similar reduction in VTE or VTE-related death and all-cause mortality, reductions in ‘major or CRNM bleed’ for initial/long-term treatment were significantly better with apixaban compared with all other NOACs, and with dabigatran compared with rivaroxaban and edoxaban. The use of NOACs represents an important step forward in the management of VTE and may therefore reduce the significant burden on patients with VTE.

## Supporting Information

S1 PRISMA Checklist PRISMA Checklist. (DOCX)

S1 Table Quality assessment of included trials. Abbreviations: ITT, intention to treat; CRNM, clinically relevant non-major; DVT, deep vein thrombosis; PE, pulmonary embolism; NA, not applicable. †Participants were not blind to treatment allocation: open label study design. Outcome events were classified by a central adjudication committee whose members were unaware of the treatment assignments.
(DOCX)

S2 Table Raw data used in NMA. Abbreviations: CRNM, clinically relevant non major; DVT, deep vein thrombosis; LMWH, low molecular weight heparin; n/A, not applicable; PE, pulmonary embolism; UFH, unfractionated heparin; VKA, vitamin K antagonist; VTE, venous thromboembolism. †Reported as events from the start of any study drug (both single- and double-dummy study period). ‡Calculated as ‘major or CRNM bleeding event’ minus ‘major bleeding event’.
(DOCX)

S3 Table Results of fixed-effect NMA—other outcomes of interest. Significant results in bold. Abbreviations: BD, twice daily; Crl, credible interval; CRNM, clinically relevant non major; OD, once daily; VKA, vitamin K antagonist; VTE, venous thromboembolism. †Defined as ‘major bleed’ minus ‘intracranial bleeding’. ‡Defined as ‘all-cause mortality minus ‘VTE-related death’ minus ‘bleeding-related death’. Data assumptions: VTE-related death was not directly reported in the trial publications and was therefore assumed based on available efficacy outcome data. For the AMPLIFY trial [21] and the EINSTEIN DVT/EINSTEIN PE pooled analysis [28] event data for this outcome were calculated from the reported incidence of PE, plus fatal events where PE could not be ruled out. For the RE-COVER [24] and RE-COVER II [22] trials it was taken as ‘death related to PE’. Event data for ‘other major bleed‘ were calculated by subtracting intracranial bleeding events from major bleeding events. This was done for event data from all trials, where available. Event data for ‘other deaths‘ were calculated by subtracting VTE- or bleeding-related deaths from all deaths.
(DOCX)

S4 Table Results of base case fixed-effect NMA–inverted treatment comparisons. Significant results in bold. Abbreviations: BD, twice daily; Crl, credible interval; CRNM, clinically relevant non major; OD, once daily; VKA, vitamin K antagonist; VTE, venous thromboembolism. †Defined as ‘major bleed’ minus ‘intracranial bleeding’. ‡Defined as ‘all-cause mortality minus ‘VTE-related death’ minus ‘bleeding-related death’.
(DOCX)

S5 Table Results of sensitivity analysis fixed-effect NMA. Significant results in bold. Abbreviations: BD, twice daily; Crl, credible interval; CRNM, clinically relevant non major; OD, once daily; PE, pulmonary embolism; VKA, vitamin K antagonist; VTE, venous thromboembolism. †Defined as ‘major bleed’ minus ‘intracranial bleeding’. ‡Defined as ‘all-cause mortality minus ‘VTE-related death’ minus ‘bleeding-related death’.
(DOCX)

S6 Table Results of sensitivity analysis fixed-effect NMA–inverted treatment comparisons. Significant results in bold. Abbreviations: BD, twice daily; Crl, credible interval; CRNM, clinically relevant non major; DVT, deep vein thrombosis; OD, once daily; PE, pulmonary embolism; VKA, vitamin K antagonist; VTE, venous thromboembolism. †Defined as ‘major bleed’ minus ‘intracranial bleeding’. ‡Defined as ‘all-cause mortality minus ‘VTE-related death’ minus ‘bleeding-related death’.
(DOCX)
