# Supplementary material for: Comparison of the Novel Oral Anticoagulants Apixaban, Dabigatran, Edoxaban, and Rivaroxaban in the Initial and Long-Term Treatment and Prevention of Venous Thromboembolism: Systematic Review and Network Meta-Analysis
Source: PLoS One. 2015 Dec 30;10(12):e0144856. doi: 10.1371/journal.pone.0144856 (PMC4696796; doi:10.1371/journal.pone.0144856)
Supplement: S1 PRISMA Checklist — (DOCX) [file pone.0144856.s001.docx]

S7 Table: PRISMA checklist

| **Section/topic** | **#** | **Checklist item** | **Reported on page #** |
| --- | --- | --- | --- |
| **TITLE** | | |  |
| Title | 1 | Identify the report as a systematic review, meta-analysis, or both. | Both: p.1 |
| **ABSTRACT** | | |  |
| Structured summary | 2 | Provide a structured summary including, as applicable: background; objectives; data sources; study eligibility criteria, participants, and interventions; study appraisal and synthesis methods; results; limitations; conclusions and implications of key findings; systematic review registration number. | Provided on p. 2 and 3 |
| **INTRODUCTION** | | |  |
| Rationale | 3 | Describe the rationale for the review in the context of what is already known. | Provided on P. 4 |
| Objectives | 4 | Provide an explicit statement of questions being addressed with reference to participants, interventions, comparisons, outcomes, and study design (PICOS). | p. 5 and Table 1 on p. 6 |
| **METHODS** | | |  |
| Protocol and registration | 5 | Indicate if a review protocol exists, if and where it can be accessed (e.g., Web address), and, if available, provide registration information including registration number. | A protocol was drafted although it was not registered on a public site. The PICOS are detailed in Table 1 on p. 6 |
| Eligibility criteria | 6 | Specify study characteristics (e.g., PICOS, length of follow-up) and report characteristics (e.g., years considered, language, publication status) used as criteria for eligibility, giving rationale. | Table 1 on p. 6 |
| Information sources | 7 | Describe all information sources (e.g., databases with dates of coverage, contact with study authors to identify additional studies) in the search and date last searched. | Provided on P. 7 |
| Search | 8 | Present full electronic search strategy for at least one database, including any limits used, such that it could be repeated. | This can be provided in a supplementary table if required for submission. |
| Study selection | 9 | State the process for selecting studies (i.e., screening, eligibility, included in systematic review, and, if applicable, included in the meta-analysis). | Provided on P. 7 |
| Data collection process | 10 | Describe method of data extraction from reports (e.g., piloted forms, independently, in duplicate) and any processes for obtaining and confirming data from investigators. | Provided on P. 7 |
| Data items | 11 | List and define all variables for which data were sought (e.g., PICOS, funding sources) and any assumptions and simplifications made. | Provided in data assumptions section on P. 8 |
| Risk of bias in individual studies | 12 | Describe methods used for assessing risk of bias of individual studies (including specification of whether this was done at the study or outcome level), and how this information is to be used in any data synthesis. | Provided on P. 7 |
| Summary measures | 13 | State the principal summary measures (e.g., risk ratio, difference in means). | Provided on P. 7 |
| Synthesis of results | 14 | Describe the methods of handling data and combining results of studies, if done, including measures of consistency (e.g., I^2^) for each meta-analysis. | Provided on P. 7 and 8 |
