# Supplementary material for: Comparison of the Novel Oral Anticoagulants Apixaban, Dabigatran, Edoxaban, and Rivaroxaban in the Initial and Long-Term Treatment and Prevention of Venous Thromboembolism: Systematic Review and Network Meta-Analysis
Source: PLoS One. 2015 Dec 30;10(12):e0144856. doi: 10.1371/journal.pone.0144856 (PMC4696796; doi:10.1371/journal.pone.0144856)
Supplement: S1 Table — Abbreviations: ITT, intention to treat; CRNM, clinically relevant non-major; DVT, deep vein thrombosis; PE, pulmonary embolism; NA, not applicable. †Participants were not blind to treatment allocation: open label study design. Outcome events were classified by a central adjudication committee whose members were unaware of the treatment assignments. (DOCX) [file pone.0144856.s002.docx]

S1 Table: Quality assessment of included trials

| **Question** | **AMPLIFY (**[**19**](#_ENREF_19)**)** | **RECOVER (**[**22**](#_ENREF_22)**)** | **RECOVER II (**[**20**](#_ENREF_20)**)** | **EINSTEIN DVT (**[**17**](#_ENREF_17)**)** | **EINSTEIN PE (**[**18**](#_ENREF_18)**)** | **Hokusai-VTE**  **(**[**21**](#_ENREF_21)**)** |
| --- | --- | --- | --- | --- | --- | --- |
|  | **Grade (yes/no/not clear/NA)** | | | | | |
| Was the method used to generate random allocations adequate? | Yes | Yes | Yes | Yes | Yes | Yes |
| Was the allocation adequately concealed? | Not clear | Yes | Not clear | Not clear | Not clear | Not clear |
| Were the groups similar at the outset of the study in terms of prognostic factors, for example, severity of disease? | Yes | Yes | Yes | Yes | Yes | Yes |
| Were the care providers, participants and outcome assessors blind to treatment allocation? | Yes | Yes | Yes | Yes/No† | Yes/No† | Yes |
| Were there any unexpected imbalances in drop-outs between groups? If so, were they explained or adjusted for? | No | No | No | No | No | No |
| Is there any evidence to suggest that the authors measured more outcomes than they reported? | No | No | No | No | No | No |
| Did the analysis include an intention-to-treat analysis? If so, was this appropriate and were appropriate methods used to account for missing data? | Yes (All efficacy analyses were based on the ITT population for whom the outcome status at 6 months was recorded. Safety analyses were conducted for the on treatment population. This was considered appropriate). | Yes (Efficacy outcomes were based on a modified ITT principle. Safety analyses were conducted for the on treatment population. This was considered appropriate). | No (The population analysed for efficacy consisted of all randomised patients who took ≥1 dose of the study drug. The safety population also consisted of all randomised patients who took ≥1 dose of study drug, but this analysis was according to the actual treatment received, and was from the first dose of trial treatment until 6 days after the trial treatment). | Yes (The primary efficacy analysis was performed on an ITT basis with the use of a stratified intended-duration Cox proportional-hazards model, adjusted for the presence of a malignant condition at baseline. The safety analysis included all patients who received the assigned study drug. This was considered appropriate). | Yes (Primary efficacy analysis was based on the ITT principle. Safety analyses were conducted for a modified ITT population (all patients who received ≥1 dose of study drug). This was considered appropriate). | Yes (Efficacy analyses were performed on a modified ITT population with stratification. Safety analyses were conducted for the on treatment population. This was considered appropriate). |

Abbreviations: ITT, intention to treat; CRNM, clinically relevant non-major; DVT, deep vein thrombosis; PE, pulmonary embolism; NA, not applicable
†Participants were not blind to treatment allocation: open label study design. Outcome events were classified by a central adjudication committee whose members were unaware of the treatment assignments
