# Supplementary material for: Comparison of the Novel Oral Anticoagulants Apixaban, Dabigatran, Edoxaban, and Rivaroxaban in the Initial and Long-Term Treatment and Prevention of Venous Thromboembolism: Systematic Review and Network Meta-Analysis
Source: PLoS One. 2015 Dec 30;10(12):e0144856. doi: 10.1371/journal.pone.0144856 (PMC4696796; doi:10.1371/journal.pone.0144856)
Supplement: S2 Table — Abbreviations: CRNM, clinically relevant non major; DVT, deep vein thrombosis; LMWH, low molecular weight heparin; n/A, not applicable; PE, pulmonary embolism; UFH, unfractionated heparin; VKA, vitamin K antagonist; VTE, venous thromboembolism. †Reported as events from the start of any study drug (both single- and double-dummy study period). ‡Calculated as ‘major or CRNM bleeding event’ minus ‘major bleeding event’. (DOCX) [file pone.0144856.s003.docx]

S2 Table: Raw data used in NMA

| **Trial** | **Trial arm** | **VTE and VTE-related death** | | **Major or CRNM bleeding** | | **Major bleeding** | | **CRNM bleeding** | | **All-cause mortality** | |
| --- | --- | --- | --- | --- | --- | --- | --- | --- | --- | --- | --- |
|  |  | **Number at risk** | **Number of events** | **Number at risk** | **Number of events** | **Number at risk** | **Number of events** | **Number at risk** | **Number of events** | **Number at risk** | **Number of events** |
| AMPLIFY ([19](#_ENREF_19)) | Apixaban | 2691 | 59 | 2676 | 115 | 2676 | 15 | 2676 | 103 | 2691 | 41 |
|  | Enoxaparin/warfarin | 2704 | 71 | 2689 | 261 | 2689 | 49 | 2689 | 215 | 2704 | 52 |
| RE-COVER ([22](#_ENREF_22)) | UFH or LMWH/Dabigatran | 1274 | 30 | 1273 | 71 | 1273 | 20 | 1273 | 51‡ | 1274 | 21 |
|  | UFH or LMWH /Warfarin | 1265 | 27 | 1266 | 111 | 1266 | 24 | 1266 | 87‡ | 1265 | 21 |
| RE-COVER II ([20](#_ENREF_20)) | UFH or LMWH/Dabigatran | 1279 | 30 | 1280 | 64 | 1280 | 15 | 1280 | 49‡ | 1279 | 25 |
|  | UFH or LMWH /Warfarin | 1289 | 28 | 1288 | 102 | 1288 | 22 | 1288 | 80‡ | 1289 | 25 |
| EINSTEIN pooled ([24](#_ENREF_24)) | Rivaroxaban | 4150 | 86 | 4130 | 388 | 4130 | 40 | 4130 | 354 | 4150 | 96 |
|  | Enoxaparin/VKA | 4131 | 95 | 4116 | 412 | 4116 | 72 | 4116 | 346 | 4131 | 99 |
| RE-COVER/ RE-COVER II pooled analysis ([20](#_ENREF_20)) | UFH or LMWH/Dabigatran | 2553 | 60 | 2553 | 136† | 2553 | 37† | 2553 | 99†, ‡ | 2553 | 46 |
|  | UFH or LMWH /Warfarin | 2554 | 55 | 2554 | 217† | 2554 | 51† | 2554 | 166†, ‡ | 2554 | 46 |
| Hokusai-VTE ([21](#_ENREF_21)) | Enoxaparin or UFH/Edoxaban | 4118 | 49 | 4118 | 349 | 4118 | 56 | 4118 | 298 | 4118 | 132 |
|  | Enoxaparin or UFH /warfarin INR 2.0-3.0 | 4122 | 59 | 4122 | 423 | 4122 | 66 | 4122 | 368 | 4122 | 126 |

Abbreviations: CRNM, clinically relevant non major; DVT, deep vein thrombosis; LMWH, low molecular weight heparin; n/A, not applicable; PE, pulmonary embolism; UFH, unfractionated heparin; VKA, vitamin K antagonist; VTE, venous thromboembolism
†Reported as events from the start of any study drug (both single- and double-dummy study period)
‡Calculated as ‘major or CRNM bleeding event’ minus ‘major bleeding event’.
