# Supplementary material for: Comparison of the Novel Oral Anticoagulants Apixaban, Dabigatran, Edoxaban, and Rivaroxaban in the Initial and Long-Term Treatment and Prevention of Venous Thromboembolism: Systematic Review and Network Meta-Analysis
Source: PLoS One. 2015 Dec 30;10(12):e0144856. doi: 10.1371/journal.pone.0144856 (PMC4696796; doi:10.1371/journal.pone.0144856)
Supplement: S3 Table — Significant results in bold. Abbreviations: BD, twice daily; Crl, credible interval; CRNM, clinically relevant non major; OD, once daily; VKA, vitamin K antagonist; VTE, venous thromboembolism. †Defined as ‘major bleed’ minus ‘intracranial bleeding’. ‡Defined as ‘all-cause mortality minus ‘VTE-related death’ minus ‘bleeding-related death’. Data assumptions: VTE-related death was not directly reported in the trial publications and was therefore assumed based on available efficacy outcome data. For the AMPLIFY trial [21] and the EINSTEIN DVT/EINSTEIN PE pooled analysis [28] event data for this outcome were calculated from the reported incidence of PE, plus fatal events where PE could not be ruled out. For the RE-COVER [24] and RE-COVER II [22] trials it was taken as ‘death related to PE’. Event data for ‘other major bleed‘ were calculated by subtracting intracranial bleeding events from major bleeding events. This was done for event data from all trials, where available. Event data for ‘other deaths‘ were calculated by subtracting VTE- or bleeding-related deaths from all deaths. (DOCX) [file pone.0144856.s004.docx]

S3 Table: Results of fixed-effect NMA - other outcomes of interest (significant results in bold)

| **Treatment comparison** | **RR (95% Crl)** | | | | | | |
| --- | --- | --- | --- | --- | --- | --- | --- |
|  | **Non-fatal PE** | **DVT** | **VTE-related death** | **Intracranial bleed** | **Other major bleed†** | **Other deaths‡** | **Overall treatment discontinuation** |
| Apixaban vs. VKA | 1.18 (0.68, 2.06) | 0.60 (0.34, 1.05) | 0.80 (0.37, 1.71) | 0.47 (0.09, 1.85) | **0.27 (0.14, 0.51)** | 0.80 (0.49, 1.32) | 0.92 (0.80, 1.04) |
| Dabigatran vs. VKA | 1.00 (0.53, 1.86) | 1.17 (0.75, 1.85) | 1.36 (0.29, 7.33) | 0.47 (0.08, 2.11) | 0.80 (0.50, 1.26) | 1.00 (0.65, 1.54) | 1.07 (0.94, 1.22) |
| Rivaroxaban vs. VKA | 1.10 (0.71, 1.71) | 0.71 (0.44, 1.11) | 1.00 (0.44, 2.23) | **0.34 (0.11, 0.90)** | **0.60 (0.39, 0.90**) | 1.02 (0.75, 1.39) | **0.84 (0.74, 0.94)** |
| Edoxaban vs. VKA | 0.83 (0.56, 1.21) | 0.90 (0.63, 1.29) | 1.00 (0.56, 1.77) | **0.26 (0.09, 0.68)** | 1.07 (0.72, 1.57) | 1.16 (0.88, 1.53) | 1.08 (0.89, 1.31) |
| Apixaban vs. dabigatran | 1.18 (0.51, 2.75) | 0.51 (0.25, 1.05) | 0.58 (0.09, 3.35) | 1.02 (0.11, 9.79) | **0.34 (0.15, 0.74)** | 0.80 (0.41, 1.55) | 0.85 (0.71, 1.03) |
| Apixaban vs. rivaroxaban | 1.08 (0.53, 2.18) | 0.86 (0.41, 1.76) | 0.80 (0.26, 2.46) | 1.40 (0.21, 8.29) | **0.46 (0.21, 0.97)** | 0.78 (0.44, 1.41) | 1.10 (0.92, 1.31) |
| Apixaban vs. edoxaban | 1.43 (0.73, 2.80) | 0.67 (0.34, 1.29) | 0.80 (0.30, 2.06) | 1.83 (0.28, 10.70) | **0.26 (0.12, 0.54)** | 0.71 (0.40, 1.27) | 0.85 (0.67, 1.07) |
| Rivaroxaban vs. dabigatran | 1.10 (0.51, 2.36) | 0.60 (0.32, 1.14) | 0.72 (0.11, 4.28) | 0.73 (0.11, 5.69) | 0.75 (0.40, 1.39) | 1.02 (0.60, 1.74) | **0.78 (0.65, 0.93)** |
| Rivaroxaban vs. edoxaban | 1.33 (0.74, 2.38) | 0.78 (0.44, 1.39) | 1.00 (0.37, 2.71) | 1.29 (0.29, 5.69) | **0.56 (0.32, 0.996)** | 0.84 (0.55, 1.28) | **0.77 (0.61, 0.97)** |
| Dabigatran vs. edoxaban | 1.21 (0.58, 2.51) | 1.29 (0.73, 2.30) | 1.37 (0.26, 7.83) | 1.38 (0.14, 9.78) | 0.75 (0.41, 1.37) | 0.87 (0.52, 1.44) | 1.00 (0.79, 1.26) |

Abbreviations: BD, twice daily; Crl, credible interval; CRNM, clinically relevant non major; OD, once daily; VKA, vitamin K antagonist; VTE, venous thromboembolism
†Defined as ‘major bleed’ minus ‘intracranial bleeding’
‡Defined as ‘all-cause mortality minus ‘VTE-related death’ minus ‘bleeding-related death’
Data assumptions: VTE-related death was not directly reported in the trial publications and was therefore assumed based on available efficacy outcome data. For the AMPLIFY trial ([19](#_ENREF_19)) and the EINSTEIN DVT/EINSTEIN PE pooled analysis ([24](#_ENREF_24)) event data for this outcome were calculated from the reported incidence of PE, plus fatal events where PE could not be ruled out. For the RE-COVER ([22](#_ENREF_22)) and RE-COVER II ([20](#_ENREF_20)) trials it was taken as ‘death related to PE’. Event data for ’other major bleed‘ were calculated by subtracting intracranial bleeding events from major bleeding events. This was done for event data from all trials, where available. Event data for ’other deaths‘ were calculated by subtracting VTE- or bleeding-related deaths from all deaths.
