# Supplementary material for: Comparison of the Novel Oral Anticoagulants Apixaban, Dabigatran, Edoxaban, and Rivaroxaban in the Initial and Long-Term Treatment and Prevention of Venous Thromboembolism: Systematic Review and Network Meta-Analysis
Source: PLoS One. 2015 Dec 30;10(12):e0144856. doi: 10.1371/journal.pone.0144856 (PMC4696796; doi:10.1371/journal.pone.0144856)
Supplement: S4 Table — Significant results in bold. Abbreviations: BD, twice daily; Crl, credible interval; CRNM, clinically relevant non major; OD, once daily; VKA, vitamin K antagonist; VTE, venous thromboembolism. †Defined as ‘major bleed’ minus ‘intracranial bleeding’. ‡Defined as ‘all-cause mortality minus ‘VTE-related death’ minus ‘bleeding-related death’. (DOCX) [file pone.0144856.s005.docx]

S4 Table: Results of base case fixed-effect NMA – inverted treatment comparisons (significant results in bold)

| **Outcome** | **RR (95% Crl)** | | | | | | | | | |
| --- | --- | --- | --- | --- | --- | --- | --- | --- | --- | --- |
|  | **VKA vs. apixaban** | **VKA vs. dabigatran** | **VKA vs. rivaroxaban** | **VKA vs. edoxaban** | **Dabigatran vs. apixaban** | **Rivaroxaban vs. apixaban** | **Edoxaban vs. apixaban** | **Dabigatran vs. rivaroxaban** | **Edoxaban vs. rivaroxaban** | **Edoxaban vs. dabigatran** |
| VTE and VTE-related-death | 1.20 (0.85, 1.70) | 0.92 (0.64, 1.32) | 1.11 (0.83, 1.49) | 1.21 (0.88, 1.68) | 1.31 (0.79, 2.16) | 1.08 (0.69, 1.69) | 0.99  (0.62, 1.59) | 1.21 (0.76, 1.93) | 0.92 (0.59, 1.42) | 0.76 (0.46, 1.23) |
| Major or CRNM bleeding | **2.27** **(1.82, 2.86)** | **1.56** **(1.28, 1.96)** | 1.06 (0.93, 1.22) | **1.21** **(1.06, 1.39)** | **1.44** **(1.07, 1.95)** | **2.13** **(1.65, 2.75)** | **1.87** **(1.45, 2.42)** | **0.68** **(0.53, 0.87)** | 0.88 (0.73, 1.07) | **1.30** **(1.01, 1.67)** |
| Major bleeding | **3.33** **(1.89, 6.25)** | 1.32 (0.85, 2.04) | **1.82** **(1.24, 2.70)** | 1.18 (0.83, 1.68) | **2.51** **(1.23, 5.34)** | 1.83 (0.92, 3.76) | **2.81** **(1.45, 5.70)** | 1.38 (0.77, 2.46) | 1.54 (0.91, 2.62) | 1.12  (0.64, 1.98) |
| CRNM bleeding | **2.08** **(1.67, 2.63)** | **1.67** **(1.32, 2.13)** | 0.98 (0.85, 1.14) | **1.24** **(1.07, 1.43)** | 1.25 (0.89, 1.75) | **2.12** **(1.62, 2.80)** | **1.68** **(1.28, 2.23)** | **0.59** **(0.44, 0.78)** | **0.79** **(0.65, 0.97)** | **1.35** **(1.02, 1.79)** |
| All-cause mortality | 1.27 (0.85, 1.92) | 1.00 (0.67, 1.49) | 1.03 (0.78, 1.37) | 0.95 (0.75, 1.22) | 1.27 (0.71, 2.26) | 1.22 (0.75, 2.01) | 1.33 (0.83, 2.14) | 1.04 (0.63, 1.69) | 1.09 (0.75, 1.57) | 1.05  (0.66, 1.69) |
| Non-fatal PE | 0.85 (0.48, 1.47) | 1.00 (0.54, 1.89) | 0.88 (0.56, 1.39) | 1.21 (0.83, 1.78) | 0.84 (0.36, 1.94) | 0.96 (0.47, 1.97) | 0.70 (0.36, 1.38) | 0.91 (0.42, 1.95) | 0.75 (0.42, 1.34) | 0.82 (0.40, 1.72) |
| DVT | 1.64 (0.95, 2.94) | 0.85 (0.54, 1.33) | 1.41 (0.90, 2.22) | 1.11 (0.78, 1.59) | 1.94 (0.95, 4.06) | 1.17 (0.57, 2.42) | 1.49 (0.78, 2.93) | 1.66 (0.87, 3.16) | 1.28 (0.72, 2.28) | 0.77 (0.43, 1.37) |
| VTE-related death | 1.25 (0.58, 2.74) | 0.73 (0.14, 3.51) | 1.01 (0.45, 2.27) | 1.00 (0.57, 1.78) | 1.71 (0.30, 10.90) | 1.25 (0.41, 3.81) | 1.25 (0.48, 3.31) | 1.37 (0.24, 8.57) | 1.00 (0.37, 2.73) | 0.73 (0.13, 3.89) |
| Intracranial bleeding | 2.13 (0.53, 11.11) | 2.13 (0.47, 12.50) | **2.94** **(1.11, 9.09)** | **3.78** **(1.48, 11.76)** | 0.99 (0.10, 8.99) | 0.72 (0.12, 4.76) | 0.55 (0.09, 3.60) | 1.08 (0.11, 7.78) | 0.78 (0.18, 3.47) | 0.72 (0.10, 6.93) |
| Other major bleeding† | **3.70** **(1.96, 7.14)** | 1.25 (0.79, 1.96) | **1.67** **(1.10, 2.56)** | 0.94 (0.64, 1.39) | **2.93** **(1.35, 6.70)** | **2.19** **(1.04, 4.87)** | **3.88** **(1.87, 8.61)** | 1.34 (0.72, 2.52) | **1.79** **(1.004, 3.17)** | 1.33 (0.73, 2.44) |
| Other deaths‡ | 1.25 (0.76, 2.06) | 1.00 (0.65, 1.54) | 0.98 (0.72, 1.34) | 0.87 (0.66, 1.14) | 1.26 (0.65, 2.42) | 1.28 (0.71, 2.29) | 1.41 (0.79, 2.52) | 1.04 (0.61, 1.76) | 1.19 (0.78, 1.82) | 1.15 (0.69, 1.93) |
| Overall treatment discontinuation | 1.09 (0.96, 1.25) | 0.93 (0.82, 1.06) | **1.19** **(1.06, 1.35)** | 0.93 (0.77, 1.12) | 1.17 (0.97, 1.41) | 0.91 (0.77, 1.09) | 1.18 (0.94, 1.49) | **1.30** **(1.08, 1.56)** | **1.30** **(1.04, 1.63)** | 1.00 (0.79, 1.27) |

Abbreviations: BD, twice daily; Crl, credible interval; CRNM, clinically relevant non major; OD, once daily; VKA, vitamin K antagonist; VTE, venous thromboembolism
†Defined as ‘major bleed’ minus ‘intracranial bleeding’
‡Defined as ‘all-cause mortality minus ‘VTE-related death’ minus ‘bleeding-related death’
