# Supplementary material for: Comparison of the Novel Oral Anticoagulants Apixaban, Dabigatran, Edoxaban, and Rivaroxaban in the Initial and Long-Term Treatment and Prevention of Venous Thromboembolism: Systematic Review and Network Meta-Analysis
Source: PLoS One. 2015 Dec 30;10(12):e0144856. doi: 10.1371/journal.pone.0144856 (PMC4696796; doi:10.1371/journal.pone.0144856)
Supplement: S5 Table — Significant results in bold. Abbreviations: BD, twice daily; Crl, credible interval; CRNM, clinically relevant non major; OD, once daily; PE, pulmonary embolism; VKA, vitamin K antagonist; VTE, venous thromboembolism. †Defined as ‘major bleed’ minus ‘intracranial bleeding’. ‡Defined as ‘all-cause mortality minus ‘VTE-related death’ minus ‘bleeding-related death’. (DOCX) [file pone.0144856.s006.docx]

S5 Table: Results of sensitivity analysis fixed-effect NMA (significant results in bold)

| **Outcome** | **RR (95% Crl)** | | | | | | | | | |
| --- | --- | --- | --- | --- | --- | --- | --- | --- | --- | --- |
|  | **Apixaban vs. VKA** | **Dabigatran vs. VKA** | **Rivaroxaban vs. VKA** | **Edoxaban vs. VKA** | **Apixaban vs. dabigatran** | **Apixaban vs. rivaroxaban** | **Apixaban vs. edoxaban** | **Rivaroxaban vs. dabigatran** | **Rivaroxaban vs. edoxaban** | **Dabigatran vs edoxaban** |
| VTE and VTE-related-death | 0.83 (0.59, 1.17) | 1.09 (0.76, 1.57) | 0.90 (0.67, 1.20) | 0.83 (0.60, 1.14) | 0.76 (0.46, 1.26) | 0.92 (0.59, 1.45) | 1.01 (0.63, 1.63) | 0.82 (0.52, 1.31) | 1.09  (0.71, 1.69) | 1.32  (0.81, 2.16) |
| Major or CRNM bleeding | **0.44** **(0.35, 0.55)** | **0.63** **(0.51, 0.77)** | 0.94 (0.82, 1.07) | **0.82** **(0.72, 0.94)** | **0.70** **(0.52, 0.95)** | **0.47** **(0.36, 0.61)** | **0.54** **(0.41, 0.69)** | **1.49** **(1.17, 1.91)** | 1.14  (0.94, 1.38) | **0.76**  **(0.60, 0.98)** |
| Major bleeding | **0.30** **(0.16, 0.53)** | 0.72 (0.47, 1.10) | **0.55** **(0.37, 0.81)** | 0.85 (0.60, 1.21) | **0.42** **(0.20, 0.84)** | 0.55 (0.27, 1.09) | **0.35** **(0.18, 0.69)** | 0.76 (0.43, 1.35) | 0.65  (0.39, 1.09) | 0.85  (0.49, 1.47) |
| CRNM bleeding | **0.48** **(0.38, 0.60)** | **0.60** **(0.47, 0.76)** | 1.02 (0.88, 1.18) | **0.81** **(0.70, 0.94)** | 0.80 (0.57, 1.12) | **0.47** **(0.36, 0.62)** | **0.59** **(0.45, 0.78)** | **1.70** **(1.29, 2.26)** | **1.26**  **(1.03, 1.55)** | **0.74**  **(0.56, 0.98)** |
| All-cause mortality | 0.79 (0.52, 1.18) | 1.00 (0.67, 1.50) | 0.97 (0.73, 1.28) | 1.05 (0.82, 1.34) | 0.79 (0.44, 1.40) | 0.82 (0.50, 1.34) | 0.75 (0.47, 1.21) | 0.96 (0.59, 1.58) | 0.92  (0.64, 1.33) | 0.92  (0.64, 1.33) |
| Non-fatal PE | 1.19 (0.68, 2.08) | 1.00 (0.52, 1.93) | 1.10 (0.71, 1.71) | 0.83 (0.57, 1.22) | 1.18 (0.50, 2.80) | 1.08 (0.53, 2.20) | 1.43 (0.73, 2.83) | 1.10 (0.50, 2.42) | 1.33 (0.74, 2.36) | 1.20 (0.56, 2.56) |
| DVT | 0.61 (0.34, 1.05) | 1.18 (0.75, 1.86) | 0.71 (0.44, 1.11) | 0.91 (0.64, 1.29) | 0.51 (0.25, 1.04) | 0.86 (0.41, 1.76) | 0.67 (0.34, 1.29) | 0.60 (0.31, 1.14) | 0.78 (0.44, 1.39) | 1.30 (0.73, 2.31) |
| VTE-related death | 0.80 (0.36, 1.72) | 0.63 (0.07, 4.12) | 0.99 (0.44, 2.24) | 1.00 (0.56, 1.78) | 1.28 (0.17, 12.29) | 0.80 (0.26, 2.48) | 0.80 (0.30, 2.08) | 1.59 (0.20, 15.73) | 1.00 (0.37, 2.67) | 0.63 (0.07, 4.45) |
| Intracranial bleeding | 0.47 (0.09, 1.87) | 0.36 (0.05, 1.77) | **0.34** **(0.11, 0.91)** | **0.26** **(0.09, 0.68)** | 1.33 (0.14, 15.45) | 1.40 (0.21, 8.35) | 1.83 (0.28, 10.70) | 0.95 (0.13, 9.23) | 1.29 (0.29, 5.69) | 1.38 (0.14, 9.78) |
| Other major bleeding† | **0.27** **(0.14, 0.51)** | 0.76 (0.49, 1.17) | **0.60** **(0.39, 0.91)** | 1.07 (0.72, 1.58) | **0.36** **(0.16, 0.77)** | **0.46** **(0.21, 0.97)** | **0.26** **(0.12, 0.53)** | 0.79 (0.43, 1.46) | **0.56** **(0.32, 0.997)** | 0.71 (0.39, 1.27) |
| Other deaths‡ | 0.80 (0.48, 1.31) | 1.05 (0.69, 1.61) | 1.02 (0.75, 1.39) | 1.16 (0.88, 1.53) | 0.76 (0.40, 1.47) | 0.78 (0.44, 1.41) | 0.71 (0.39, 1.25) | 0.97 (0.57, 1.65) | 0.84 (0.55, 1.28) | 0.91 (0.54, 1.50) |

Abbreviations: BD, twice daily; Crl, credible interval; CRNM, clinically relevant non major; OD, once daily; PE, pulmonary embolism; VKA, vitamin K antagonist; VTE, venous thromboembolism
†Defined as ‘major bleed’ minus ‘intracranial bleeding’
‡Defined as ‘all-cause mortality minus ‘VTE-related death’ minus ‘bleeding-related death’
